# Correction of defective CFTR/ENaC function and tightness of cystic fibrosis airway epithelium by amniotic mesenchymal stromal (stem) cells

**DOI:** 10.1111/jcmm.12303

**Published:** 2014-06-03

**Authors:** Annalucia Carbone, Stefano Castellani, Maria Favia, Anna Diana, Valentina Paracchini, Sante Di Gioia, Manuela Seia, Valeria Casavola, Carla Colombo, Massimo Conese

**Affiliations:** a Fondazione IRCCS Ca' Granda Ospedale Maggiore Policlinico, Medical Genetics LaboratoryMilan, Italy; b Department of Medical and Surgical Sciences, University of FoggiaFoggia, Italy; c Department of Biosciences, Biotechnologies and Biopharmaceutics, University of BariBari, Italy; d Cystic Fibrosis Regional Center, Azienda Ospedaliero-Universitaria PoliclinicoBari, Italy; e Fondazione IRCCS Ca' Granda Ospedale Maggiore Policlinico, Cystic Fibrosis CenterMilan, Italy

**Keywords:** amniotic membrane, cell therapy, mesenchymal stromal cells, actin, tight junctions, CFTR, ENaC

## Abstract

Cystic fibrosis (CF) is caused by mutations in the CF transmembrane conductance regulator (CFTR) gene, with most of the mortality given by the lung disease. Human amniotic mesenchymal stromal (stem) cells (hAMSCs) hold great promise for regenerative medicine in the field of lung disease; however, their potential as therapeutics for CF lung disease has not been fully explored. In the present study, hAMSCs were analysed in co-cultures on Transwell filters with CF immortalized airway epithelial cells (CFBE41o- line) at different ratios to exploit their potency to resume basic defects associated with CF. The results show that F-actin content was increased in co-cultures as compared with CF cells and actin was reorganized to form stress fibres. Confocal microscopy studies revealed that co-cultures had a tendency of increased expression of occludin and ZO-1 at the intercellular borders, paralleled by a decrease in dextran permeability, suggestive of more organized tight junctions (TJs). Spectrofluorometric analysis of CFTR function demonstrated that hAMSC-CFBE co-cultures resumed chloride transport, in line with the appearance of the mature Band C of CFTR protein by Western blotting. Moreover, hAMSC-CFBE co-cultures, at a 1:5 ratio, showed a decrease in fluid absorption, as opposed to CFBE cell monolayers that displayed a great rate of fluid resorption from the apical side. Our data show that human amniotic MSCs can be used in co-culture with CF respiratory epithelial cells to model their engraftment into the airways and have the potential to resume a tight epithelium with partial correction of the CF phenotype.

## Introduction

Cystic fibrosis (CF) is a lethal autosomal recessive disorder as a result of mutations in the CF transmembrane conductance regulator (CFTR) gene, a cAMP-dependent chloride channel expressed on the apical side of epithelial cells [1]. Although CF involves many organs with secretory/absorptive properties, the main cause of morbidity and mortality is a chronic inflammatory lung disease. The basic defect in CF airway epithelium is given by a dual defect in chloride secretion, as a result of the lack/dysfunction of CFTR, and in sodium absorption, because of the hyperactivity of the epithelial sodium channel (ENaC), through a still unidentified regulatory mechanism. When CFTR is defective, an increased ENaC-mediated Na^+^ absorption [2–4] leads to a reduction of the periciliary fluid volume with a consequent collapse of the mucous layer entrapping the cilia [2]. The consequent impairment of the mucociliary clearance would favour the bacterial colonization of the airways and a chronic inflammatory response.

Because of its monogenic nature, and as the lung is easily accessible, CF has been a target disease for gene-based therapeutic intervention; however, this approach has given unsatisfactory results in terms of efficiency of gene delivery to the lung and of efficacy outcome [5]. This partial success was because of the inefficiency of passing the mucus barrier overlying the epithelial cells and the immune response against the gene therapy vectors [6]. Cell therapy could be a more effective treatment because allogeneic normal cells and autologous engineered cells express CFTR gene. Indeed, various cell types have been utilized for treating CF [7]. Bone marrow (BM)-derived stem cells have been the first source evaluated for homing to the lung and curative potential. Wang and colleagues transduced CF BM-MSCs with a lentiviral vector bearing a wtCFTR gene and, upon co-culturing them with CF airway epithelial cells, obtained a resumption of the chloride efflux [8]. However, the use of BM has some limitations, including the low frequency of MSCs and the invasive procedure for obtaining them. Moreover, the age and disease state may affect the collection of sufficient healthy autologous BM for transplantation [9–11]. Finally, expansion of autologous BM cells could represent a cumbersome and low-yield approach, and, last but not the least, the *in vivo* efficiency of BM stem cells to differentiate in airway epithelium is very low (0.01–0.025%) [12], as also demonstrated by different studies in CF mice [13,14].

Recently, we have identified and preliminarily characterized in the context of CF a new cell source, derived from the placenta, *i.e*. human mesenchymal stromal (stem) cells (hAMSCs) displaying features of stemness and potential differentiation towards hepatocyte-like cells and airway epithelial cells. In co-cultures of hAMSCs and CF respiratory epithelial cells, we demonstrated that hAMSCs can acquire CFTR expression at the level of mRNA and protein, an effect probably induced by the contact with CF cells, as this effect was not observed in separate co-cultures [15].

In this paper, we sought to study the effect on the basic defect in CF by co-culturing hAMSCs with CF airway epithelial cells homozygous for commonest mutation in these patients, *i.e*. F508del. According to the current hypothesis in CF pathogenesis of the lung disease, we demonstrate that hAMSCs can increase the F508del CFTR-dependent chloride secretion and diminish the fluid hyper-absorption. Furthermore, we also analysed whether co-cultures could recover other defects recognized in CF cells, *i.e*. actin fibres and tight junction disorganization.

## Materials and methods

### Isolation and culture of human amniotic mesenchymal stromal (stem) cells

Human amniotic mesenchymal stromal (stem) cells (hAMSCs) were isolated from term placentas (*n* = 3), which would normally be discarded after delivery. Tissues were obtained under appropriate approval from the Ethical Committee of Fondazione IRCCS Ca' Granda Ospedale Maggiore Policlinico (Milan) and signed informed consent. All the procedures followed the Declaration of Helsinki protocols. All infectious pathogen-positive deliveries, including those involving HBV, HCV and HIV, as well as cases of pre-diagnosed genetic abnormalities, were excluded. Placenta samples were procured immediately after delivery and processed under sterile conditions. After peeling from the placenta and washing with calcium- and magnesium-free HBSS (CMF-HBSS, Lonza, Treviglio, Italy) supplemented with 0.5 mM EGTA (Sigma-Aldrich, Milan, Italy), amnion membranes were processed to remove epithelial cells as previously reported [16]. Once epithelial cells were removed, the amniotic membranes were digested to collect hAMSCs [17]. Briefly, amniotic membranes were washed three times with cold HBSS, cut into pieces and transferred into 50-ml centrifuge tubes; about 30–40 ml of digestion solution composed of EMEM (Lonza) supplemented with 25 mM HEPES buffer without L-glutamine (Lonza), 1 mg/ml collagenase type IV and 25 μg/ml DNase I (both from Sigma-Aldrich). Membranes were incubated on a rotator between 45 min. and 1.5 hrs, depending on tissue thickness, at 37°C. After blocking the enzymatic reaction with cold HBSS, cell suspensions were centrifuged two times for 5 min. at 200 × g, 4°C and counted by using a Bürker chamber. After isolation, DNA was obtained from hAMSCs by phenol/chlorophorm extraction. Purified DNA was investigated for most frequent mutations in CFTR gene by using the commercial kit (Inno-Lipa CFTR19, Inno-Lipa CFTR17+ TnUpdate, Inno-Lipa CFTR-Italian Regional – Innogenetics, Ghent, Belgium).

Cells were plated at a density of 1 × 10^5^ cells/cm^2^ in standard culture medium composed of DMEM (Lonza) supplemented with 1% sodium pyruvate, 10% (v/v) heat-inactivated foetal bovine serum (FBS), 1% non-essential amino acid, 55 μM β-mercaptoethanol (all by Invitrogen, Milan, Italy), 1% L-glutamine, 1% antibiotics solution (both by Cellgro, Manassas, VA, USA) and 10 ng/ml epidermal growth factor (EGF; Sigma-Aldrich), according to the previously reported protocol [17]. Medium was replaced 2 hrs after plating to remove unattached contaminating epithelial cells and then every 2 days. Each batch of hAMSCs was characterized for stemness and mesenchymal antigens by flow cytometry, as previously described [15].

### Cell cultures

Experiments were performed in four human immortalized bronchial epithelial cell lines. Three of them, 16HBE14o-, expressing wild-type CFTR; CFBE41o- bearing F508del CFTR, homozygous for the F508del allele; CFBE/wtCFTR, CFBE41o- cells stably expressing wild-type CFTR, were a generous gift of Professor D. Gruenert (University of California at San Francisco, USA). CFBE/wtCFTR cells were maintained in presence of 200 μg/ml hygromycin B-positive selection. The CFBE41o- cells, stably overexpressing F508del CFTR (CFBE-F508del), were a generous gift from Dr. J.P. Clancy, University of Cincinnati, Children's Hospital Medical Center, Ohio, USA) [18]. CFBE-F508del were grown in complete media (E-MEM, 10% FBS, L-glutamine and penicillin/streptomycin) in presence of 2 μg/ml puromicin-positive selection at 37°C under 5% CO_2_. Cells were routinely grown on flasks coated with an extracellular matrix containing fibronectin/vitrogen/BSA. Epithelial cells were grown in complete medium, alone or in co-culture with hAMSCs. H441 (Human lung adenocarcinoma epithelial cells) cells were grown in RPMI 1640 medium containing 10% FBS, 4.5 g/l glucose, 100 U/ml penicillin and 100 μg/ml streptomycin.

Passage two-hAMSCs were mixed with CFBE41o- cells at different ratios (1:5, 1:10, 1:20) and, to obtain polarized co-cultures, cells were seeded on 6.5-mm diameter Snapwell, 0.4-μm pore size (Corning, Acton, MA, USA) at 1 × 10^5^ per filter coated with a solution of 10 μg/ml Fibronectin (BD Biosciences, San Jose, CA, USA), 100 μg/ml albumin from bovine serum (Sigma-Aldrich), and 30 μg/ml bovine collagen type I (BD Biosciences) dissolved in MEM. As controls, hAMSCs and CFBE41o- cells were seeded at 2.5 × 10^4^ and 1 × 10^5^ per filter respectively. Co-cultures were maintained in the CFBE cell culture medium at 37°C under 5% CO_2_ for at least 6–8 days. In some immunofluorescence experiments, hAMSCs were labelled with chloromethylbenzamido (CellTracker™ CM-DiI) [15,19].

Separate co-cultures were obtained by seeding hAMSCs onto the filter and CFBE41o- cells onto the bottom of the lower chamber. To obtain 1:5 and 1:10 ratios, hAMSCs were seeded at 2.0 × 10^4^ and 1.0 × 10^4^ and CFBE41o- cells at 8.0 × 10^4^ and 9.0 × 10^4^ respectively. Medium was changed daily for 5 days, and cultures were analysed at day 6.

### Real-time RT-PCR

Total RNA was isolated from freshly isolated and cultured cells with TRIZOL® Reagent (Invitrogen), according to the manufacturer's protocol. One microgram of RNA was reverse-transcribed into first-strand cDNA with the High-Capacity cDNA Reverse Transcription kit (Applied Biosystems, Life Technologies Italia, Monza, Italy) by using random primers following manufacturer's instructions.

To evaluate ENaC (α, β and γ subunits) and CFTR expression, a SYBR Green-based real-time PCR assay using the comparative method (on Mx3005P Stratagene instrument, La Jolla, CA, USA) was carried out. β-actin was used as endogenous control (normalizer). PCRs were set up in triplicate in a total volume of 25 μl per capillary. One reaction mixture contained 12.5 μl of Brilliant SYBR® Green QPCR Master Mix from Stratagene, including a SureStart® Taq DNA polymerase, a dNTP mixture and SYBR Green, MgCl_2_ (2.5 mM), 0.1 μl of forward and reverse primers each (final concentration: 200 nM), 0.375 μl of diluted reference dye (final concentration: 30 nM), 1 μl cDNA and 10.925 μl H_2_O. The calibrator was cDNA obtained from H441 cells treated with dexamethasone (50 nM for 6 hrs) for ENaC amplification and cDNA obtained from 16HBE14o- cells for CFTR amplification. The cycling conditions were as follows: an initial activation step of 95°C for 10 min. followed by 40 cycles of denaturation of 95°C for 30 sec., annealing at 57°C for 1 min. and extension at 72°C for 30 sec. Primer sequences of amplifications are reported in Table S1.

### Analysis of F-actin content

Actin polymerization assay was performed as previously described [20,21]. Briefly, cells in co-culture, grown on coated 35-mm dishes, were fixed with 3.7% formaldehyde and permeabilized in 0.1% Triton X-100 in PBS. The cells were then incubated with 0.25 mM Phalloidin-TRITC in buffer containing 20 mM KH_2_PO_4_, 2 mM MgCl_2_, 5 mM EGTA and 10 mM PIPES (pH 6.8 with KOH) for 1 hr. Cells were incubated in methanol at 4°C overnight to extract phalloidin linked to F-actin. After extraction, the cells were washed with PBS and a Bradford Coomassie Plus Protein Assay (Pierce, Thermo Fisher Scientific Inc., Rockford, IL, USA) was performed to determine total cell protein content. Fluorescence emission at 565 nm on excitation at 540 nm was measured with a Cary Eclipse plate reader (Varian, Palo Alto, CA, USA). Fluorescence emission values were normalized to protein levels for each sample.

### Immunofluorescence and confocal analysis

Cells grown on filters were washed three times with PBS, and incubated in 2% BSA in PBS for 30 min. on ice. Cells were incubated with FITC-conjugated anti-ZO-1 or anti-occludin antibodies diluted 1:20 in 0.2% BSA in PBS for 1 hr on ice. After two washes in PBS, cells were fixed in 3% PFA, 2% sucrose for 10 min. In separate experiments, cells were stained with 20 nM phalloidin-TRITC to visualize F-actin. After three washes in PBS, filters were excised and placed side up on a glass slide, and overlaid with a drop of Mowiol (Calbiochem, San Diego, CA, USA) followed by a coverslip. Cells were analysed by using a Nikon TE2000 microscope coupled to a Radiance 2100 confocal dual-laser scanning microscopy system (Bio-Rad, Segrate, Italy). Specimens were viewed through a 60× oil immersion objective, with a 2.5 zooming in some analyses. Digital images were processed by using the program Laser Sharp 2000 (Bio-Rad). XY planes are shown at 5–6 slices from the apical level.

### hAMSC labelling and CFTR cytofluorimetric assay

Passage two-hAMSCs were labelled with chloromethylbenzamido (CellTracker™ CM-DiI) [19]. Stock solutions of CM-DiI were prepared in dimethylsulfoxide (DMSO) at 1 ng/μl. Immediately before labelling, the stock solution was diluted up to a final concentration of 0.005 ng/μl in DMEM without phenol red. Cells grown at confluence in a T25 flask were washed with PBS and then incubated with the dye working solution for 30 min. at 37°C. After labelling, cells are washed twice with PBS, then incubated at 37°C 5% CO_2_ for at least 24 hrs in the presence of fresh medium.

Co-cultures at 3, 6 and 12 days were analysed for CFTR expression by flow cytometry, as previously performed [15]. Briefly, cells were detached and incubated with CFTR antibody MAB25031 mouse IgG2a (R&D Systems, Minneapolis, MN, USA) used at 1:20 dilution for 1 hr at 4°C. After washing in PBS, the cells were incubated with the FITC-conjugated secondary antibody (anti mouse used at 1:100; Sigma-Aldrich) for 1 hr at 4°C, followed by two washes in PBS, and analysed. As a background control, co-cultures were incubated with secondary antibody only, and the resulting fluorescence was subtracted from the analysed samples incubated both with primary and secondary antibodies. Data were collected by using a Coulter Epix XL flow cytometer (Beckman Coulter, Fullerton, CA, USA). Ten thousand cells were examined in each experiment. As physical parameters (forward scatter and side scatter) did not allow us to distinguish hAMSCs from CFBE41o- cells, specific expression of CFTR on hAMSCs was detected in the CM-DiI-labelled cells. Analysis was performed by plotting the FLH-1 channel (525 nm), identifying the CFTR-specific green signal, against the FLH-2 channel (575 nm), identifying the red-labelled hAMSCs. The vitality was evaluated by trypan blue exclusion assay and resulted to be >98%.

### Protein extraction and Western blotting

After 7 days of co-culture on permeable filter inserts, cells were washed with PBS, homogenized in lysis buffer (NaCl 110 mM, Tris 50 mM, Triton X-100 0.5%, and Igepal CA-630 0.5%, pH 8.0, with added protease inhibitor mixture), sonicated for 10 sec. and centrifuged for 10 min. (16,000 × g), and then the pellet was discarded. Supernatant protein concentration was measured, and an aliquot of 30 μg of protein was diluted in Laemmli buffer, heated at 100°C for 5 min. and separated by 3–8% Tris-acetate gel (Bio-Rad). The gel was transferred to polyvinylidene difluoride membranes (GE Healthcare Italia, Milan, Italy) and processed for Western blotting by using monoclonal CFTR antibody (R&D systems, MAB25031; dilution 1:500) or monoclonal ß-tubulin (Sigma-Aldrich; dilution 1:1000). The secondary antibody was anti-mouse IgG for both primary antibodies (Sigma-Aldrich). Immunocomplexes were detected with LumiGLO reagent (Cell Signaling, EuroClone, Milan, Italy) and densitometric quantification and image processing were carried out by using Adobe Photoshop and the Image software package (version 1.61, National Institutes of Health, Bethesda, MD, USA).

### Fluorescence measurements of apical chloride efflux

Chloride efflux was measured by using the Cl^−^-sensitive dye, *N*-(Ethoxy-carbonylmethyl)-6-methoxyquinolinium bromide (MQAE) as we previously reported [22]. After 7 days of co-culture on permeable filter inserts, cells were loaded overnight in culture medium containing 5 mM MQAE at 37°C in a CO_2_ incubator and then inserted into a perfusion chamber that allowed independent perfusion of apical and basolateral cell surfaces. Fluorescence was recorded with a Cary Eclipse spectrofluorometer (Varian). To measure chloride efflux rate across the apical membrane, the apical perfusion medium was changed with a medium in which chloride was substituted with iso-osmotic nitrate. All experiments were performed at 37°C in HEPES-buffered bicarbonate-free media (Cl^−^ medium: 135 mM NaCl, 3 mM KCl, 1.8 mM CaCl_2_, 0.8 mM MgSO_4_, 20 mM HEPES, 1 mM KH_2_PO_4_, 11 mM glucose, and Cl^−^ free-medium: 135 mM NaNO_3_, 3 mM KNO_3_, 0.8 mM MgSO_4_, 1 mM KH_2_PO_4_, 20 mM HEPES, 5 mM Ca(NO_3_)_2_ and 11 mM glucose). We measured the apical CFTR-dependent chloride secretion as described previously: [22] CFTR-dependent chloride secretion was calculated as the difference in the rate of change of FSK-plus 3-isobutyl-1-methylxanthine (IBMX)-stimulated fluorescence in the absence or presence of apical treatment with the specific CFTR inhibitor, CFTRinh-172 [23,24].

### Dextran permeability

CFBE41o-, 16HBE14o-, hAMSCs, and co-cultures of hAMSCs with CFBE41o- at different ratios, were seeded on a 6.5-mm diameter Transwell at 1 × 10^5^ per filter and grown for 5–6 days. Then, FITC-conjugated dextrans of different molecular weight (10 kD [10s] and 2000 kD [2000s]; Sigma-Aldrich) were added to the apical side of monolayers and after 10, 30 and 50 min. the apparent permeability (pAPP) was calculated by measuring the fluorescence in the basal medium as previously described [25].

### Apical fluid re-absorption

Transepithelial fluid transport measurement was performed according to Aarbiou *et al*. [26]. Briefly, the apical surface of epithelia was washed with a saline solution containing (in mM): 137 NaCl, 2.7 KCl, 8.1 Na_2_HPO_4_, 1.5 KH_2_PO_4_, 1 CaCl_2_ and 0.5 MgCl_2_. The apical medium was removed, then 200 μl of saline solution at room temperature was added to the apical surface. Filters were rotated gently to remove the medium remaining at the walls of the cup, and then the fluid was recovered. This process was repeated three times. After washing, the apical side of the epithelium was covered with 50 μl of the same saline solution (with or without 30 μM camostat mesylate, Sigma-Aldrich), and 150 μl of mineral oil to prevent evaporation. Cells were maintained at 37°C in 5% CO_2_ with complete medium present in the basolateral compartment. After 24 hrs, the apical fluid was carefully removed, centrifuged to separate the mineral oil, and the residual volume of aqueous phase was measured. Fluid absorption is reported as ml/(cm^2^ h).

### Statistical analysis

Statistical significance of differences was evaluated by a two-tailed unpaired Student's *t*-test. Data were analysed by using Prism 4 (GraphPad Software, Inc., La Jolla, CA, USA). *P* values of less than 0.05 were considered significant.

## Results

A desirable outcome of stem cell administration would be the engraftment within the airway epithelium to orchestrate the correction of the CF basic defect. As an *in vitro* surrogate model for *in vivo*, we studied co-cultures of hAMSCs with immortalized CFBE41o- (CFBE) cells bearing the most frequent mutation in CF, *i.e*. F508del. After having demonstrated that hAMSCs used in this study were not bearing any CF mutations (data not shown), in the first part of our study, we have focused on the morphological and functional aspects of CF cells related to actin stress fibre formation and TJ organization and gate function.

### Co-cultures show an increased content of F-actin and induce its reorganization in stress fibres

As shown in Figure 1A, CFBE cells displayed substantial disorganization of actin filaments as previously described [21], whereas in hAMSC-CFBE co-cultures cells we noticed an increase in cortical actin stress fibre formation, which was appreciable as a staining between the cell borders. In particular, the hAMSC-CFBE co-cultures at 1:5 ratio resembled the organization found in control 16HBE14o- (16HBE) cells expressing wild-type CFTR (Fig. 1F). Interestingly, the less hAMSCs in the culture, *i.e*. at 1:10 and 1:20, the less the actin reorganization was observed (Fig. 1C and D). hAMSCs displayed long actin fibres diffused in their cytoplasm (Fig. 1E), as previously described [27]. It is possible also to observe that the reorganization of actin happened in CFBE cells and not in hAMSCs, as these cells are characterized by spindle-like form and bigger nuclei. A higher magnification of cells show a detailed organization of actin filaments at the cell borders in 1:5 hAMSC-CFBE co-cultures, which was similar to the pattern of 16HBE cells, whereas the staining with 1:10 co-cultures was less evident, but nevertheless present at the cell–cell contacts (Fig. S1).

**Fig. 1:**
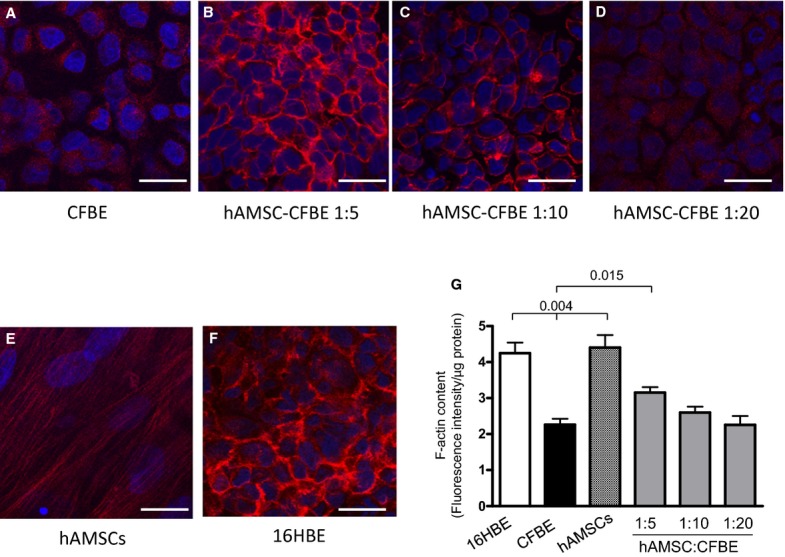
Co-cultures show a reorganization of actin cytoskeleton. CFBE cells (**A**), hAMSC-CFBE co-cultures at 1:5 (**B**), 1:10 (**C**), 1:20 (**D**) ratios, hAMSCs (**E**) and 16HBE cells (**F**) grown on Transwells for 6 days were stained with TRITC-phalloidin for F-actin and counterstained with DAPI for the nuclei; scale bar = 20 μm. F-actin content was obtained in the same culture conditions, detected by an actin polymerization assay and expressed as fluorescence intensity normalized to protein content of each sample (**G**). Data are expressed as mean ± SEM of three experiments.

These morphological data were supported by the quantitative analysis of F-actin content. As shown in Figure 1G in agreement with the results obtained by confocal analysis, both 16HBE cells and hAMSCs had a significantly higher F-actin content than that of CFBE cells. However, CFBE cells in co-culture with hAMSCs at a 1:5 ratio showed a significant increase in F-actin content in comparison with CFBE cells cultured alone. Interestingly, this co-culture-dependent increase in F-actin was not observed in co-cultures at 1:10 and 1:20 ratios.

### Co-cultures induce a reorganization of tight junctions and increase of the epithelial barrier function

We recently showed that CF airway epithelial cells display a defect in both tight junction organization and function as compared with non-CF epithelia [28]. In particular, ZO-1 and occludin were expressed at lower levels and did not localize at intercellular borders. As shown in Figure 2, while 16HBE cells expressing wild-type CFTR (positive control) showed a typical chicken wire pattern of ZO-1 localization at the intercellular lining (Fig. 2A), the CFBE cells, as previously found, display a lack of ZO-1 expression (Fig. 2B). hAMSCs cultured alone on filters did not display any detectable ZO-1 expression (Fig. 2C), while in hAMSC-CFBE co-cultures at 1:5 and 1:10, either ZO-1 expression or its localization at intercellular borders of CFBE cells was increased (Fig. 2D and E). Co-cultures at 1:20 did not display any difference in ZO-1 expression and localization as compared with CFBE cells alone (Fig. 2F). Higher magnification confirmed that a partial ZO-1 reorganization appeared at intercellular borders in 1:5 and 1:10 hAMSC-CFBE co-cultures, as compared with no staining in CFBE alone and 1:20 ratio (Fig. S2). Interestingly, a more reorganized pattern of ZO-1 was observed in CF cells in close contact with a single mesenchymal cell stained with CM-Dil (Fig. S3), suggesting the importance of the physical interaction among the hAMSC and the CFBE cells in inducing the rescue of the TJ protein organization. The pattern of occludin expression in both 16HBE and CFBE cells was identical to that observed for ZO-1 (Fig. S4). Also, the analysis of co-cultures at different ratios showed the same distribution for occludin and ZO-1, that is at the intercellular borders for 1:5 and 1:10 ratios and more diffuse for 1:20 ratio (Fig. S4).

**Fig. 2:**
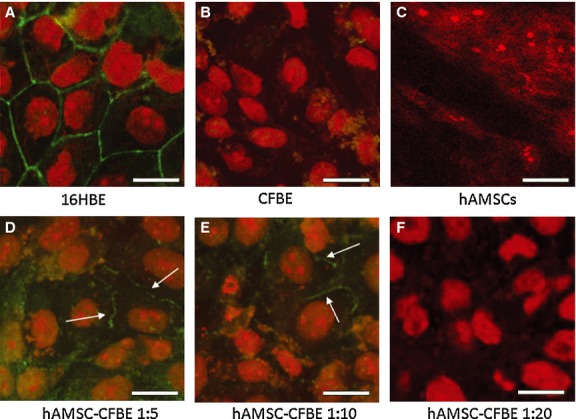
Co-cultures display a reorganized pattern of ZO-1. 16HBE (**A**), CFBE (**B**), hAMSCs (**C**), hAMSC-CFBE co-cultures at 1:5 (**D**), 1:10 (**E**), 1:20 (**F**) ratios, grown on Transwells for 6 days were stained with FITC-conjugated anti-ZO-1 antibody and counterstained with propidium iodide for nuclei. Intercellular ZO-1 pattern is indicated by white arrows; scale bar = 10 μm.

Dextran permeability allows assessing the barrier function of TJs [25]. Here, we used two FITC-conjugated dextrans, the 10s (10,000 kD) and the 2000s (2,000,000 kD), which on the basis of their molecular weight should appreciate subtle differences in the barrier function of TJs. CFBE cells showed a significantly higher permeability in comparison with 16HBE cells with both dextrans, confirming a lower organization of TJs (Fig. 3A and B), whereas co-cultures (in particular at 1:5 and 1:10 ratios) displayed a significant reduction in dextran permeability. Although the dextran 2000s crosses the epithelium with more difficulty, however, it was possible to demonstrate a significant different behaviour of co-cultures in comparison with CFBE cells (Fig. 3B). To appreciate whether the physical contact was necessary to induce the resumption of the TJ barrier function, hAMSCs and CFBE cells were grown separately on the filter and on the bottom of the Transwell respectively. The effect of decreased paracellular permeability observed with direct co-cultures was not obtained with indirect co-cultures (Fig. 3C and D) by using both dextrans, indicating again that contact between cells is at the basis of increased TJ barrier function.

**Fig. 3:**
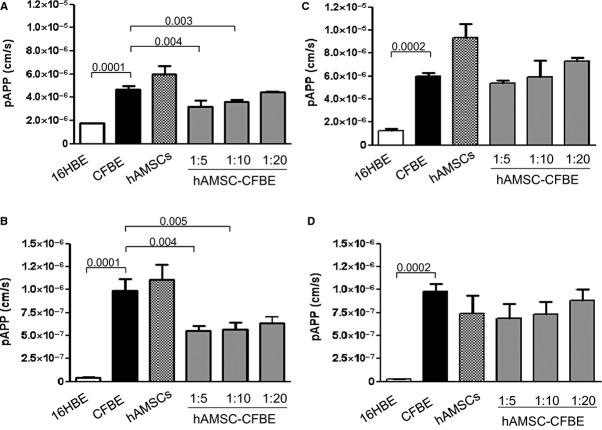
Co-cultures determine a decrease in paracellular permeability to dextrans. FITC-dextrans, 10s (**A** and **C**) or 2000s (**B** and **D**), were added to the apical side of monolayers and after 10, 30 and 50 min., the apparent permeability (pAPP) was calculated by measuring the fluorescence in the basal medium. Results obtained in direct co-cultures are shown in (**A**) and (**B**), while those obtained in indirect co-cultures are shown in (**C**) and (**D**). Data are expressed as mean ± SEM of three experiments.

### hAMSCs acquire CFTR expression in co-culture in a time-dependent fashion

CFTR plasma membrane expression was studied by a flow cytometric assay that we have already set up, demonstrating that after 6 days of co-culture, hAMSCs acquired ∼50% of CFTR expression with a 1:5 hAMSC-CFBE ratio over a 6% of background in hAMSCs alone [15]. To understand whether this expression was time-dependent, CFTR signal was detected in co-cultures at different time-points. As shown in Table 1, there was a significant increase in CFTR expression in hAMSC-CFBE co-cultures at 1:5 ratio already at 3 days (33.0 ± 6.0% *versus* 4.5 ± 0.4% of positive cells). CFTR expression in hAMSCs at day 6 confirmed previous results and tended to a further increase after 12 days of co-culture. CFTR expression in CFBE cells increased after 6 and 12 days of culture on Transwells as compared with day 3 (both *P* < 0.001). To investigate whether this effect of co-cultures was obtained with another CF cell line, the same experiment at day 6 with different hAMSC-CFBE cell ratios was carried out with CFBE cells overexpressing the F508del mutation. Table 2 shows that it was possible to detect a significant increase of CFTR-specific signal in CM-DiI-labelled cells at all hAMSC-CFBE-F508del ratios as compared with hAMSCs cells alone. The 1:5 ratio determined the higher CFTR expression in hAMSCs. Overall, these data show that a population of hAMSCs with low CFTR expression has increased this expression upon co-cultures with CF epithelial cells, also in the presence of overexpression of the CFTR-F508del mutation.

**Table 1:** Percentages of CFTR+ hAMSCs labelled with CM-DiI in co-cultures with CFBE41o- cells

	3			6			12		
			
Days of co-culture	CFBE	hAMSCs	1:5 hAMSC-CFBE	CFBE	hAMSCs	1:5 hAMSC-CFBE	CFBE	hAMSCs	1:5 hAMSC-CFBE
% of CM-Dil+ CFTR+	3.7 ± 0.3	4.5 ± 0.4	10.6 ± 2.0	10.2 ± 0.4*	5.8 ± 0.4	17.9 ± 0.5	11.8 ± 0.7**	3.8 ± 1.1	21.5 ± 0.05
% of CFTR+ in whole CM-Dil+ population		4.5 ± 0.4	33 ± 6.0**		5.8 ± 0.4	53.0 ± 5.2***		3.8 ± 1.1	64.5 ± 0.6***

Percentages of CM-DiI^+^CFTR^+^ cells were obtained by plotting the FLH-1 channel, identifying CFTR-specific green signal, against FLH-2 channel, identifying red-labelled hAMSCs. Percentages of CFTR-expressing hAMSCs in whole CM-DiI^+^ population were obtained by dividing the double-positive hAMSCs for all CMDiI^+^ cells (with and without green signal). As hAMSCs are stained with CM-Dil, the same percentage is present in both rows. Data are shown as the mean ± SEM of three experiments. Day 3: **P < 0.01, 1:5 hAMSC-CFBE *versus* hAMSCs. Day 6: *P < 0.05, CFBE *versus* hAMSCs; ***P < 0.001, 1:5 hAMSC-CFBE *versus* hAMSCs. Day 12: **P < 0.01, CFBE *versus* hAMSCs; ***P < 0.001, 1:5 hAMSC-CFBE *versus* hAMSCs.

**Table 2:** Percentages of CFTR+ hAMSCs labelled with CM-DiI in co-cultures with CFBE-F508del cells

	% of CM-DiI^+^ CFTR^+^ cells	% of CFTR^+^ in whole CM-DiI^+^ population	*P*
hAMSCs	5.5 ± 0.8	5.5 ± 0.8	_
CFBE-F508del	_	15.5 ± 0.5	0.0004
hAMSC-CFBE 1:5	26.4 ± 1.7	42.0 ± 6.3	0.0046
hAMSC-CFBE 1:10	18.3 ± 0.1	30.3 ± 7.4	0.0284
hAMSC-CFBE 1:20	18.2 ± 0.2	34.6 ± 9.3	0.0351

Percentages of CM-DiI^+^CFTR^+^ cells were obtained by plotting the FLH-1 channel, identifying CFTR-specific green signal, against FLH-2 channel, identifying red-labelled hAMSCs. Percentages of CFTR-expressing hAMSCs in whole CM-DiI^+^ population were obtained by dividing the double-positive hAMSCs for all CMDiI^+^ cells (with and without green signal). As hAMSCs are stained with CM-Dil, the same percentage is present in both columns. Data are shown as the mean ± SEM of three experiments. Significance is referred to CFTR^+^ cells in the whole CM-DiI^+^ population in all conditions as compared with hAMSCs alone.

### Co-cultures show resumption of CFTR maturation and activity

In the second part of the study, we focused on the functional behaviour of co-cultures in regard to CFTR and ENaC expression and activity. We compared the expression of CFTR mRNA in CFBE cells and hAMSCs in relation to its expression in 16HBE cells. CFTR mRNA was found to be expressed at low levels in CFBE cells as compared with 16HBE cells (Fig. S5). hAMSCs expressed even lower levels than CFBE cells at the isolation (T0), which decreased further at passage 1 (P1), compatible with our previous results at the protein level [15].

CFTR protein expression was further investigated by Western blotting analysis in co-cultures. CFTR is a glycoprotein that exits from the endoplasmic reticulum as 160 kD immature form (Band B) and then is further glycosylated to a 180 kD mature form (Band C), which is finally inserted in the plasma membrane. Figure 4A shows a typical Western Blot performed in 16HBE cells expressing wt CFTR, CFBE cells stably overexpressing wtCFTR (CFBE/wtCFTR), CFBE cells alone or in co-culture at different ratios of hAMSC-CFBE (1:5 and 1:20). In 16HBE and CFBE/wtCFTR cells, the mature Band C of CFTR is strongly expressed, whereas, on the contrary, in the CFBE cells, F508del CFTR is almost completely expressed as the immature Band B. In the co-cultures (particularly evident in 1:5 ratio), there was both the appearance of the mature Band C and an increase of immature Band B expression of F508del CFTR protein. Figure 4B shows a histogram summarizing the densitometric analysis values of Western Blots of three independent experiments, where the results for mature Band C of F508del CFTR are normalized to values of densitometry of β-tubulin band. Densitometry analysis revealed that 1:5 co-cultures rescued significantly the mature band C of the F508del CFTR with respect to the values observed in CFBE41o- cells. These data are compatible with the detection of a CFTR-specific signal in hAMSCs when co-cultured with CFBE cells [15].

**Fig. 4:**
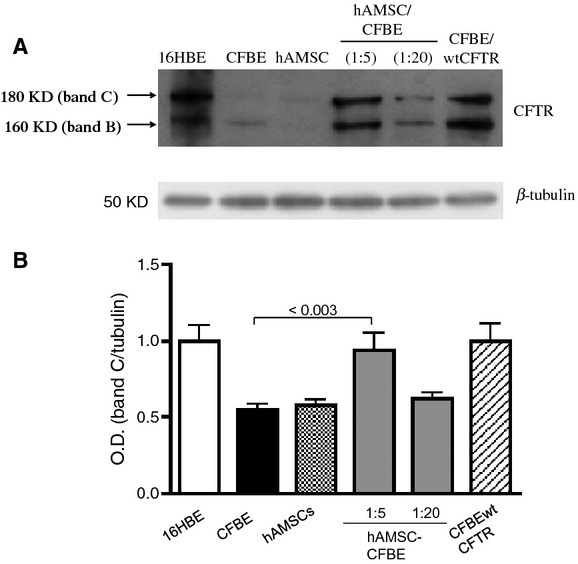
Co-cultures are characterized by a corrected CFTR protein expression. (**A**) Representative Western blot of a typical experiment of CFTR protein expression in hAMSC-CFBE co-cultures. The lysates of 16HBE and CFBE/wtCFTR are shown in the first and last lane, respectively, as a reference to the location of Band C and Band B of wtCFTR. The same membrane was probed with anti β-tubulin to confirm that protein loading was the same across the gel. (**B**) The histogram summarizes the relative change in the expression of Band C in co-culture with respect to CFTR protein Band C expression in 16HBE cells normalized as 1. Results represent means ± SEM of three independent experiments.

These results were supported by the functional analysis of CFTR-dependent chloride efflux. After 7 days of co-culture of CFBE cells with hAMSCs on permeable filters, the rate of chloride efflux was measured by following the change in fluorescence ((F/F_0_)/min) of the chloride-sensitive dye MQAE, after replacement of chloride by nitrate in the apical perfusion medium as previously described [22]. The apical F508del CFTR-dependent chloride secretion was calculated as the difference in alterations of 10 μM FSK plus 500 μM IBMX-stimulated fluorescence in absence or presence of apical treatment with 5 μM CFTR_Inh_-172. As shown in Figure 5A, CFTR-dependent chloride efflux was significantly rescued in hAMSCs grown in co-culture with CFBE in a ratio-dependent fashion. The ratio 1:5 hAMSC-CFBE produced a maximum level of rescue, with CFTR-dependent chloride efflux values resembling those of 16HBE cells. It is also possible to note that the rescue was dependent on the hAMSC-CFBE ratio, being lower with less hAMSCs in the co-culture. Although indirect co-cultures showed a significant increase in CFTR activity with respect to CFBE cells, these values were not significantly different from values of CFTR activity obtained in hAMSCs grown alone ( Fig. 5A).

**Fig. 5:**
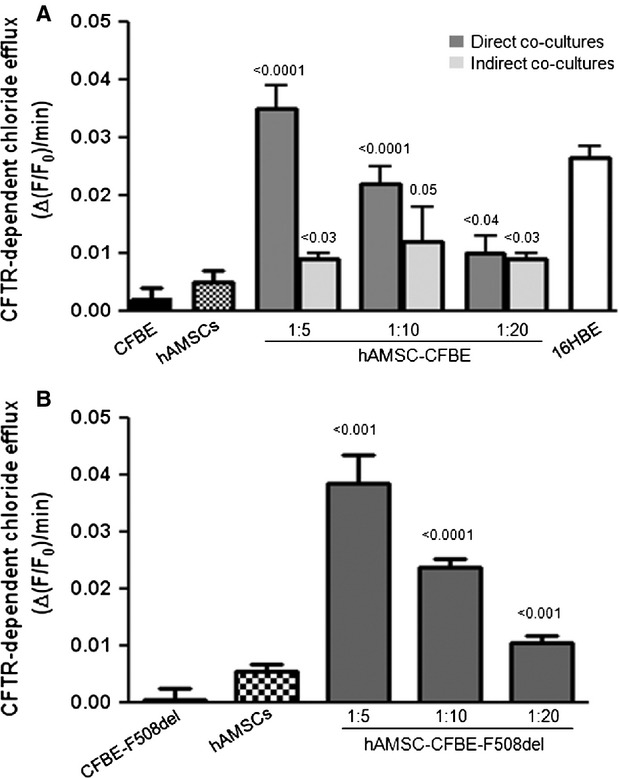
Co-cultures show resumption of CFTR-dependent chloride efflux. (**A**) CFTR-dependent chloride efflux was evaluated in 16HBE, CFBE, hAMSCs and hAMSC-CFBE at different ratios (1:5, 1:10 and 1:20) obtained both in direct and separate co-cultures. Data are expressed as mean ± SEM of 4–9 independent experiments. Statistical comparison was made with respect to the values obtained in CFBE cells. (**B**) CFTR-dependent chloride efflux was evaluated in CFBE-F508del, hAMSCs and hAMSC-CFBE-F508del at different ratios (1:5, 1:10 and 1:20) in direct co-cultures. Data are expressed as mean ± SEM of five independent experiments. Statistical comparison was made with respect to the values obtained in CFBE-F508del cells.

Because of the positive results obtained in the CFTR cytofluorimetric assay, we investigated also the behaviour of co-cultures in regard to the rescue of chloride efflux in the presence of CFBE cells overexpressing F508del CFTR (CFBE-F508del), which are extensively used in physiological studies of F508del CFTR activation [29,30]. Figure 5B shows a clear ratio-dependent rescue of CFTR-dependent chloride secretion, confirming the resumption of chloride efflux defect with another CF cell line.

### Co-cultures decrease the fluid hyper-absorption phenotype

As co-cultures resumed the defect in CFTR-dependent chloride efflux, we looked at the fluid absorption, as this is considered one of the main basic pathophysiological issues in the early steps of CF. First, we analysed mRNA ENaC subunit expression. H441 cells show increased expression of ENaC subunits upon dexamethasone challenge, as we previously showed [26], and thus dexamethasone-induced H441 was used as a calibrator for this assay. CFBE cells show a greater β subunit mRNA content (Fig. S5) and a lower α subunit mRNA content (Fig. S5) than 16HBE cells, while γ subunit looked to have similar levels in 16HBE and CFBE cells. hAMSCs showed negligible levels of all three subunits (Fig. S5).

CFBE epithelia showed a significant increase of apical fluid absorption as compared with 16HBE monolayers (Fig. 6A). Camostat, an inhibitor of proteases involved in ENaC channel activation [31], significantly diminished the fluid absorption in both 16HBE and CFBE cell monolayers. hAMSCs presented a fluid absorption rate intermediate between that of 16HBE and CFBE, which was not influenced by camostat (Fig. 6A). When fluid absorption was assessed in direct hAMSC-CFBE co-cultures, we were able to appreciate a reduction in fluid absorption at all ratios as compared with CFBE cells, although it can be seen that in the 1:20 cell co-culture, the fluid absorption rate approached that of CFBE (Fig. 6B). Indirect co-cultures showed a significant reduction in the fluid absorption rate only at the 1:5 ratio (Fig. 6C).

**Fig. 6:**
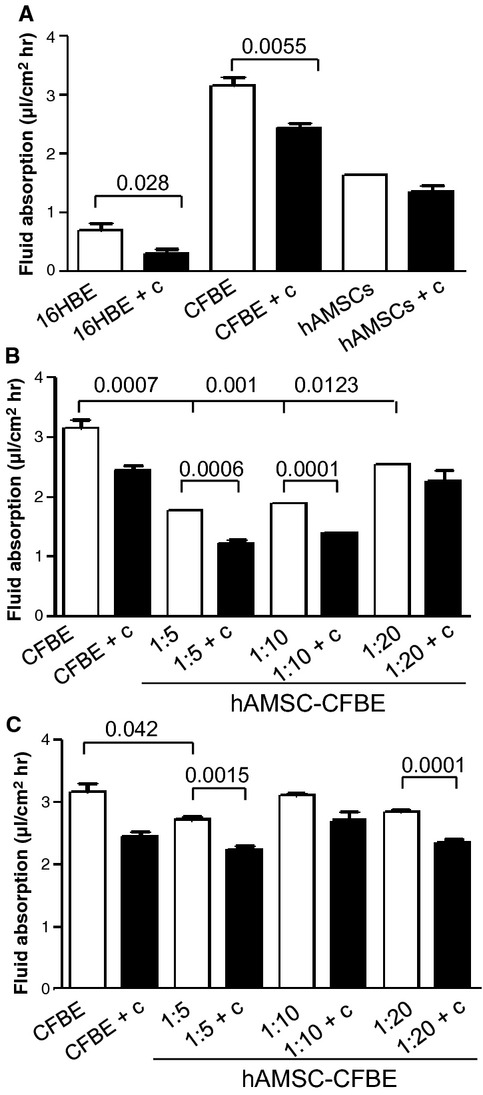
Co-cultures show a reduction in fluid hyper-absorption phenotype. Apical fluid re-absorption was studied after incubation for 24 hrs in the presence or absence of camostat (30 μM). (**A**) 16HBE, CFBE and hAMSCs were grown on Transwells for 6 days and assayed for fluid absorption for further 24 hrs in the presence or absence of camostat (c). (**B**) Direct co-cultures at the ratios of 1:5, 1:10 and 1:20 were studied as in (**A**). (**C**) Indirect co-cultures at the ratios of 1:5, 1:10 and 1:20 were studied as in (**A**).

## Discussion

Human MSCs are pluripotent stem cells initially identified in post-natal BM in 1976 by Friedenstein *et al*. [32], but have now been identified in numerous tissues, including lung, umbilical cord, cord blood, adipose tissue and placenta [33,34]. MSCs have the potency to differentiate into mesodermic cell types, such as adipocytes, osteocytes and chondrocytes *in vitro*. Further studies have shown their capacity to give rise to differentiated cell types belonging to ectodermic and endodermic lineages, including airway epithelial cells. In the last years, it has been recognized by numerous studies that MSCs might provide the lung microenvironment with paracrine mediators, which act on the different cellular structural components of the lung, *i.e*. epithelial cells, fibroblasts and endothelial cells, with cytoprotective effects, and other factors with inflammatory and immunomodulatory capacities. Among the various sources of MSCs, we have focused our attention on the amniotic membrane [35], an easily accessible and ethically acceptable source, as the term placenta is discarded after delivery. In the present study, we exploited the usefulness of hMSCs in correcting the basic defects associated with CF.

In this study, immortalized cell lines were used. We acknowledge the importance of primary airway cell cultures, although this source is limited because of the paucity of material obtainable from patients. The creation of immortalized cell lines has allowed proceeding with biochemical and pharmacological studies [36,37]. Indeed, immortalized cell lines have been shown to display a proper phenotype [38] and to give comparable results with primary airway epithelial cells as regarding chloride efflux in the presence of CFTR correctors [39,40], endoplasmic reticulum stress, intracellular Ca^++^ and response to inflammatory mediators [41,42]. With this caveat in mind, our data demonstrate the partial correction of four basic defects associated with CF in airway epithelial cells: (*i*) actin cytoskeletal disorganization; (*ii*) tight junction disorganization; (*iii*) reduced chloride transport; and (*iv*) excessive apical fluid absorption. Although not completely understood, these four alterations could be related to each other. In epithelial cells, the NHERF (Na^+^/H^+^ exchanger regulatory factor) family of scaffolding proteins has been identified as a link between CFTR and the actin-based cytoskeleton. The COOH terminus of CFTR can bind through a PDZ-binding motif to NHERF1 and NHERF2, which in turn can bind to members of the ERM family of proteins, such as ezrin, ultimately bridging CFTR to the actin cytoskeleton [21]. These interactions at the apical region of epithelial cells are thought to assemble CFTR into apical signal complexes and to regulate its function [43]. The interplay between CFTR, ENaC and actin can be by direct or indirect binding *via* actin-binding proteins [44,45]. Furthermore, the inhibition of ENaC, and thus the regulation of sodium and fluid transport across the airway epithelium, is dependent also on these correct interactions [46]. We have previously shown that overexpression of CFTR or NHERF1 in CFBE cells restored the actin cytoskeleton organization and CFTR chloride channel activity [21]. Likewise, lack of appropriate structure and function of TJs in CF cells is resumed by overexpression of CFTR or NHERF1 [28]. hAMSCs interacting with CFBE cells determined a partial reorganization of the actin cytoskeleton and structure and function of TJs, as demonstrated by confocal microscopy and dextran permeability respectively. Both ZO-1 and occludin accumulated on the intercellular borders. hAMSCs seemed to direct this effect as they were found in close contact with a cluster of CF airway epithelial cells.

In parallel with actin and TJ reorganization, we observed that CFTR protein was expressed by hAMSCs upon co-culture with epithelial cells for 6 days over the baseline (hAMSCs alone), as demonstrated unequivocally by flow cytometry, and in keeping with previously published results [15]. The acquisition of CFTR expression by hAMSCs was obtained also in co-culture with a CFBE cell line overexpressing F508del mutation, suggesting this phenotypic modulation as a more general phenomenon. Furthermore, CFTR expression by hAMSCs was found to be ratio- and time-dependent (Tables 1 and 2). Already at day 3 of co-culture, ∼30% of hAMSCs presented CFTR plasma membrane expression, indicating that the induction begins at short times of cell–cell contact and is stabilized at longer times (up to 12 days). Overall, together with those results showing the lack of induction in separate co-cultures [15], these data strongly indicate that cell-to-cell contacts are necessary for the induction of the epithelial-like phenotype in hAMSCs. We are currently studying whether other epithelial markers, particularly of the airway epithelium, are expressed by hAMSCs during the co-culture.

In association with these results, rescue of chloride channel activity along with the appearance of the band C associated with a mature fully glycosylated CFTR protein was observed (Figs 4 and 5A). These data are compatible with those previously demonstrating that 50% hAMSCs in co-culture expressed CFTR on their plasma membrane ([15] and present work). It is noteworthy that the rescue of chloride efflux was achieved also with CFBE cells overexpressing F508del mutation (Fig. 5B), which is indicative of the capacity of hAMSCs to correct this CF-associated defect in CF cells expressing higher levels of the mutated F508del CFTR.

The fluid absorption data show, as expected, that CFBE cells are endowed with an hyper-absorptive phenotype, which may be dependent on the lack of wtCFTR on the apical plasma membrane and presence instead of F508del CFTR, which is not able to inhibit ENaC activity [47]. Interestingly, however, CFBE-associated fluid hyper-absorption could be inhibited by amiloride ([26] and data not shown) and also by camostat, indicating that a protease-sensitive ENaC is expressed by CF cells. Camostat, an inhibitor of prostasin, a trypsin-like serine protease involved in activation of ENaC, is being investigated as an experimental drug in CF patients to inhibit sodium transport in the nose, restore hydration of the airway mucus and then contrast the pathophysiological cascade in this deadly disease [48]. hAMSCs presented an intermediate phenotype as concerning fluid hyper-absorption between CFBE and 16HBE cells, but in this case, camostat (and amiloride) did not exert any effect, suggesting that other channels may be at work in these cells when grown on filters. It is known that fluid absorption from the airways is dependent from different channels, including, for example, Na-K-ATPase [49]. There are some studies suggesting that MSCs can influence the absorptive behaviour of respiratory epithelial cells and thus be involved in fluid clearance from the lung [50], therefore further investigation will be conducted to better understand MSC behaviour at the molecular level. Nevertheless, it is worth noting that the correction of the fluid hyper-absorptive phenotype was achieved with all the hAMSC-CFBE ratios, indicating that this process is as well sensitive to MSC-based therapy as that associated with chloride transport. Conversely, previous gene therapy studies demonstrated that 25% of corrected cells are necessary to obtain a resumption of chloride transport [51], while the correction of fluid hyper-absorption needed nearly 100% of corrected cells [52–54]. Under the experimental conditions of co-cultures, we deal with a more complex system, based on cell-to-cell interactions and presumably paracrine factors, than the gene transfer studies mentioned above.

Murphy and colleagues [55] found that human amniotic epithelial cells (hAECs) expressed the CFTR gene and protein after extended culture (28 days) in Small Airway Growth Medium (SAGM). They also observed that hAECs, induced to express CFTR, possessed functional iodide/chloride ion channels that were inhibited by the CFTR inhibitor CFTR-172, indicating the presence of functional CFTR ion channels. The induction of CFTR expression in our previous [15] and present study was much faster than that observed in hAECs in SAGM but is consistent with the induction of surfactant protein gene expression by hAECs *in vivo* [56]. This suggests that differentiation of placental cells, either hAMSCs or hAECs, into lung lineages may be most efficient when in direct contact with lung cells as would happen with *in vivo* engraftment. If this is the case then partial differentiation *in vitro* before administration into the airway as a therapy for CF may not be necessary.

Our previous study [15] indicated that a direct contact between hAMSCs and CFBE cells is necessary to obtain a significant increase of CFTR-specific signal in hAMSCs. This was also suggested by the work by Wang *et al*. [8]. We confirm this hypothesis in the present work where we show that CFTR-associated chloride transport was enhanced in separate co-cultures but with lower efficacy. Since the cellular interactions between epithelial and mesenchymal cells in monolayer co-culture are likely to be bi-directional, a possible mode of action could be cross talk between cells *via* gap junctions, which has been observed *in vivo* in the lung between transplanted MSCs and resident epithelial cells [57]. Moreover, other mechanisms of communication between epithelial and mesenchymal stem cells are being unrevealed. Aliotta and colleagues demonstrated that lung-derived microvesicles are consumed by marrow cells inducing the expression of lung-specific genes in marrow cells, augmenting their capacity of becoming epithelial cells upon transplantation [58]. Both direct transfer of pulmonary epithelial cell-specific mRNA to marrow and induced transcription of pulmonary epithelial cell-specific mRNA in marrow cells were suggested [59]. Finally, direct horizontal transfer of mitochondria has been shown *in vivo* [60]. On the other hand, hAMSCs are also capable to secrete factors involved in regulation of inflammation and immune response, which we have not studied in this work. However, our study was not finalized to comprehend paracrine factors and mechanisms of intercellular communication, which is the focus of ongoing studies.

## Conclusion

In summary, co-cultures can be used to model the engraftment of hAMSCs into the airway epithelium and to correct the basic defects in CF airway epithelial cells. hAMSCs are an ethically acceptable source for cells to be used in regenerative medicine and thus hold promise for the treatment of genetic and acquired diseases of the lung.

## Supporting information

**Figure S1** Detailed reorganization of actin cytoskeleton in hAMSC-CFBE co-cultures.

**Figure S2** Detailed ZO-1 staining in hAMSC-CFBE cell co-cultures.

**Figure S3** Co-cultures of labelled hAMSCs and CFBE cells show a partial reorganization of ZO-1 at the level of CFBE cells.

**Figure S4** Reorganization of occludin in co-cultures.

**Figure S5** CFTR and ENaC mRNA expression.

**Table S1** Sequence of primers used in this study.
