# Supplementary figures and images for: Correction of defective CFTR/ENaC function and tightness of cystic fibrosis airway epithelium by amniotic mesenchymal stromal (stem) cells
Source: J Cell Mol Med. 2014 Jun 3;18(8):1631–43. doi: 10.1111/jcmm.12303 (PMC4190909; doi:10.1111/jcmm.12303)

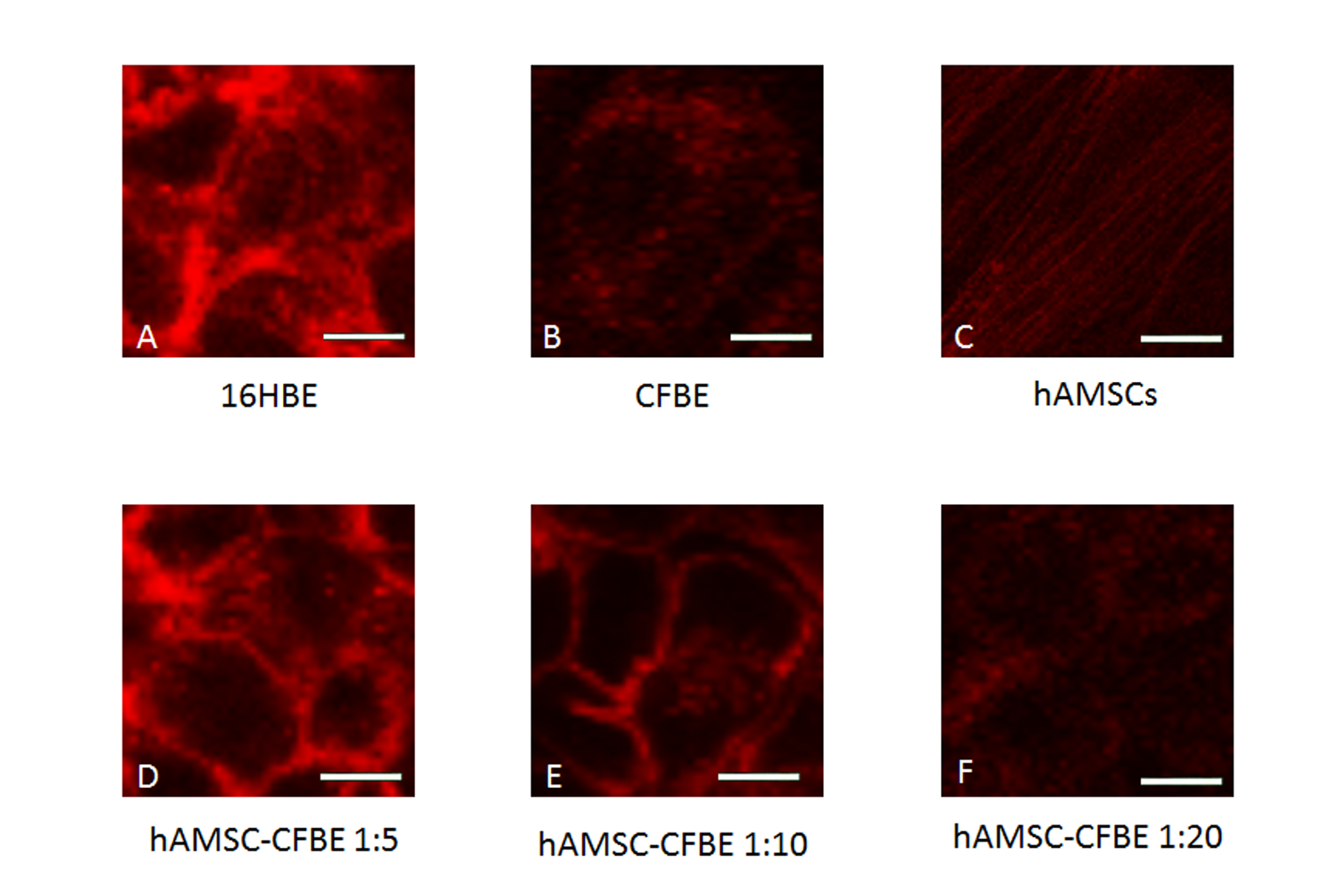

Supplement: Supplementary file 1 — Figure S1 Detailed reorganization of actin cytoskeleton in hAMSC-CFBE co-cultures. [file jcmm0018-1631-SD1.tif]

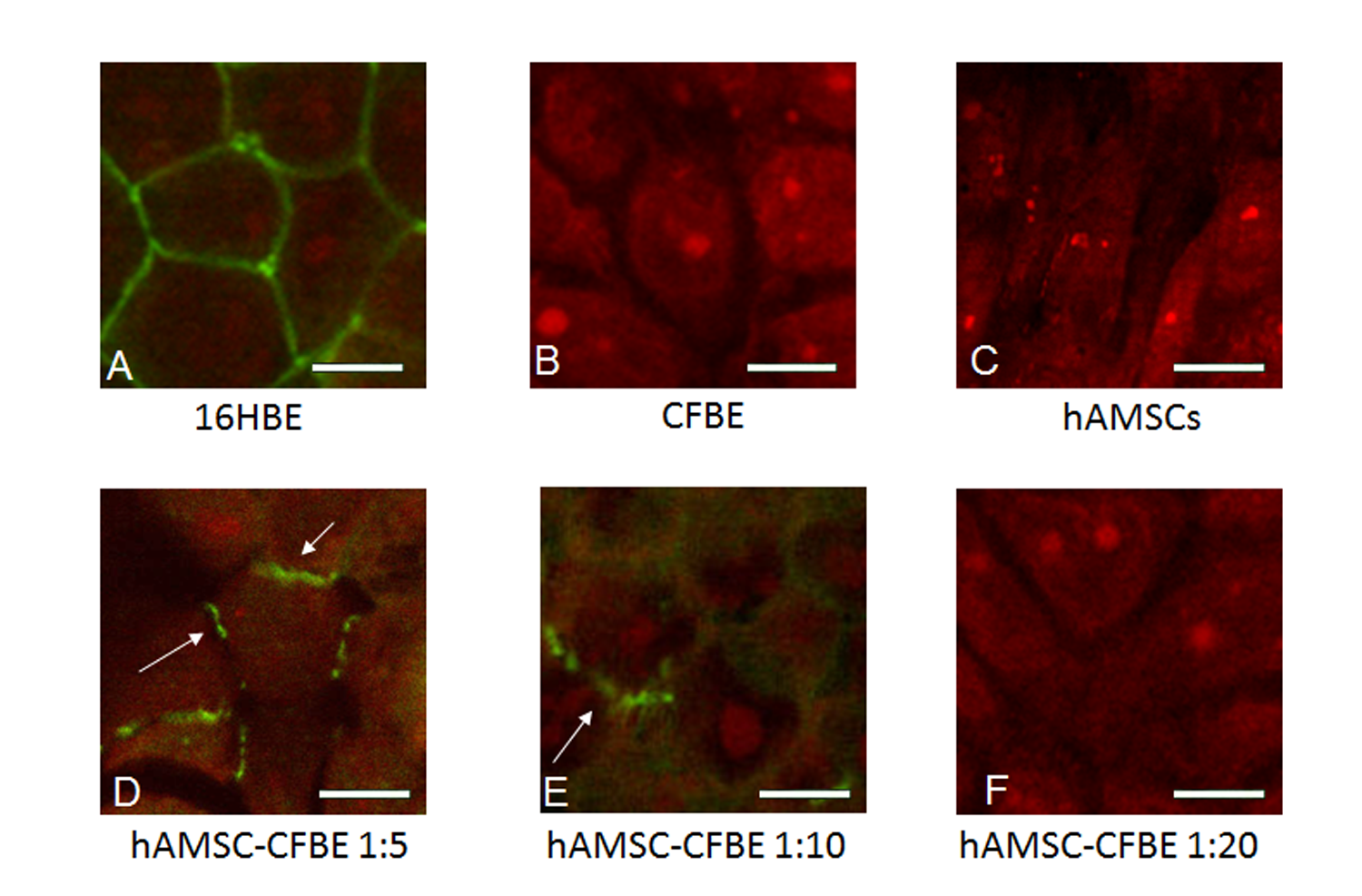

Supplement: Supplementary file 2 — Figure S2 Detailed ZO-1 staining in hAMSC-CFBE cell co-cultures. [file jcmm0018-1631-SD2.tif]
