# Supplementary material for: Correction of defective CFTR/ENaC function and tightness of cystic fibrosis airway epithelium by amniotic mesenchymal stromal (stem) cells
Source: J Cell Mol Med. 2014 Jun 3;18(8):1631–43. doi: 10.1111/jcmm.12303 (PMC4190909; doi:10.1111/jcmm.12303)
Supplement: Supplementary file 6 — Table S1 Sequence of primers used in this study. [file jcmm0018-1631-SD6.docx]

**Table S1**. Sequence of primers used in this study.

| Gene | Ref. | Exon | Primer forward (5’🡪 3’) | Primer reverse (5’🡪 3’) |
| --- | --- | --- | --- | --- |
| ENaC α | [^1^](#_ENREF_1) | 1 | CTTTGGCATGATGTACTGGCA | GGAAGACGAGCTTGTCCGAGT |
| ENaC β | [^2^](#_ENREF_2) | 2 | ACCAGGAACCTGAACTTCTCC | GTGGTTGTCTCCAAAGAGATCAA |
| ENaC γ | [^1^](#_ENREF_1)^,^ [^2^](#_ENREF_2) | 2 | GCCCTGAAGTCCCTGTATGG | CTGCTTTCCCTCTGAGACGG |
| CFTR | Home made | 17 b | GCAGCCTTACTTTGAAACTC | TAACAGCAATGAAGAAGATGAC |
| β-actin | [^3^](#_ENREF_3) | 5 | GCTCCTCCTGAGCGCAAG | CATCTGCTGGAAGGTGGACA |

REFERENCES

1 Song W, Liu G, Bosworth CA, et al. Respiratory syncytial virus inhibits lung epithelial Na+ channels by up-regulating inducible nitric-oxide synthase. J Biol Chem*.* Mar 13 2009;284(11):7294-7306.

2 Xu H, Chu S. ENaC alpha-subunit variants are expressed in lung epithelial cells and are suppressed by oxidative stress. Am J Physiol Lung Cell Mol Physiol*.* Dec 2007;293(6):L1454-1462.

3 Kamphuis W, Schneemann A, van Beek LM, Smit AB, Hoyng PF, Koya E. Prostanoid receptor gene expression profile in human trabecular meshwork: a quantitative real-time PCR approach. Invest Ophthalmol Vis Sci*.* Dec 2001;42(13):3209-3215.
