# Supplementary material for: Correction of defective CFTR/ENaC function and tightness of cystic fibrosis airway epithelium by amniotic mesenchymal stromal (stem) cells
Source: J Cell Mol Med. 2014 Jun 3;18(8):1631–43. doi: 10.1111/jcmm.12303 (PMC4190909; doi:10.1111/jcmm.12303)
Supplement: Supplementary file 7 — Table S1 Sequence of primers used in this study. [file jcmm0018-1631-SD7.doc]

**Figure S1** Detailed re-organization of actin cytoskeleton. 16HBE cells (**A**), CFBE (**B**), hAMSCs (**C**), hAMSC-CFBE co-cultures at 1:5 (**D**), 1: 10 (**E**), 1: 20 (**F**) ratios, grown on Transwells for 6 days were stained with TRITC-phalloidin for F-actin. Images were obtained by zooming 2.5X the pictures shown in Figure 1. For clarity of staining, DAPI staining was omitted. Scale bar = 5 µm.

**Figure S2** Detailed ZO-1 staining in hAMSC:CFBE cell co-cultures. 16HBE (**A**), CFBE (**B**), hAMSCs (**C**), hAMSC-CFBE co-cultures at 1:5 (**D**), 1: 10 (**E**), 1: 20 (**F**) ratios, grown on Transwells for 6 days were stained with FITC-conjugated anti-ZO-1 antibody and counterstained with propidium iodide for nuclei. Intercellular ZO-1 pattern is indicated by white arrows. Original magnification was 60X with zooming 2.5X. Scale bar = 5 μm.

**Figure S3** Co-cultures of labelled hAMSCs and CFBE cells show a partial re-organization of ZO-1 at the level of CFBE cells. hAMSCs were labelled with CM-DiI , co-cultured with unlabelled CFBE cells for 6 days, and stained with FITC-conjugated anti-ZO-1 antibody. ZO-1 organization was studied in 16HBE (**A**), CFBE (**B**) and co-cultures at 1:5 (**C**) and 1:20 (**D**). Scale bar = 10 μm.

**Figure S4** Re-organization of occludin in co-cultures**.** 16HBE (**A**), CFBE (**B**), hAMSCs (**C**), hAMSC-CFBE co-cultures at 1:5 (**D**), 1: 10 (**E),** 1: 20 (**F**) ratios**,** grown on Transwells for 6 days were stained with FITC-conjugated anti-occludin antibody and counterstained with propidium iodide for nuclei. Intercellular occludin pattern is indicated by white arrows. Scale bar = 10 μm.

**Figure S5** CFTR and ENaC mRNA expression**.** CFTR (**A**), α ENaC (**B**), β ENaC (**C**), and γ ENaC (**D**) mRNA levels were studied by Real-Time PCR. In (**A**) the CFTR expression is relative to that of 16HBE which was set at 1. In (**B**, **C**, and **D**) the ENaC subunit mRNA expression is relative to that of dexamethasone-treated H441 cells.
